# The interplay of coping styles and optimism/pessimism in shaping mental health in long-term survivors of malignant melanoma: a register-based cohort study

**DOI:** 10.1186/s40359-025-02704-1

**Published:** 2025-04-12

**Authors:** Judith Hirschmiller, Tamara Schwinn, Sabine Fischbeck, Ana Nanette Tibubos, Jörg Wiltink, Rüdiger Zwerenz, Sylke R. Zeissig, Elmar Brähler, Manfred E. Beutel, Mareike Ernst

**Affiliations:** 1 Department of Psychosomatic Medicine and Psychotherapy, University Medical Center of the Johannes Gutenberg-University Mainz, Untere Zahlbacher Str. 8, 55131 Mainz, Germany; 2 Department of Nursing Science, Diagnostic in Healthcare and eHealth, University of Trier, Trier, Germany; 3 University Cancer Center Mainz (UCT), University Medical Center of the Johannes Gutenberg-University Mainz, Mainz, Germany; 4 Institute of Clinical Epidemiology and Biometry (ICE-B), Julius-Maximilians University of Würzburg, Würzburg, Germany; 5 Regional Centre Würzburg, Bavarian Cancer Registry, Bavarian Health and Food Safety Authority, Würzburg, Germany; 6 Department of Medical Psychology and Medical Sociology, University Medical Center Leipzig, Leipzig, Germany; 7 Department of Clinical Psychology, Psychotherapy and Psychoanalysis, Institute of Psychology, University of Klagenfurt, Klagenfurt am Wörthersee, Austria

**Keywords:** Anxiety, Cancer, Coping, Depression, Optimism, Protective factors, Psycho-oncology

## Abstract

**Background:**

Optimism and pessimism are stable, overarching dispositions that influence mental health, especially in stressful life situations, such as cancer survival. They have been associated with more specific coping strategies. This study sought to investigate a theoretically-based model of their interplay in shaping depressive and anxiety symptoms to inform prevention and intervention efforts.

**Methods:**

The registry-based study included 689 survivors of malignant melanoma. We assessed sociodemographic and disease-related variables, optimism/pessimism (LOT-R), coping strategies (BC), depressive (PHQ-9), and anxiety symptoms (GAD-7). A structural equation model was conducted to analyse the hypothesized associations, modelling coping strategies (denial/self-blame, seeking external support, active coping) as mediators of the relationship of optimism/pessimism with depressive and anxiety symptoms. As a sensitivity analysis, gender-stratified models were tested.

**Results:**

The proposed model fit the data well. In the full sample, optimism was directly related to depression and anxiety, and the effects of optimism and pessimism were mediated via denial/self-blame. This indirect effect accounted for 60.8% of the total effect of pessimism on depression, and for 79.55% on anxiety. Stratified analyses showed different patterns of associations by gender, in the sense that the mediation effect was more relevant among men.

**Conclusion:**

This study shows the relevance and need of gender-sensitive psychosocial-care. Especially in men, psychosocial interventions should target maladaptive coping strategies. Within women, fostering optimism seems to be particularly important. As the model did not fit as well for women, more gender-sensitive research is needed to understand potentially different risk/protective factors and needs of support.

**Supplementary Information:**

The online version contains supplementary material available at 10.1186/s40359-025-02704-1.

## Trial registration

The study procedure and materials were approved by the Ethical Committee of the Statutory Physician Board of the State Rhineland Palatinate (Reference number 837.161.11 (7703)).

**Supplementary Information:**

The online version contains supplementary material available at 10.1186/s40359-025-02704-1.

## Background

Malignant melanoma is one of the most common cancer diagnoses in Germany (in women 4th and in men the 5th most common type of cancer) [1], showing an increased incidence in most countries (possibly due to increased risk factors and demographic changes) [2]. However, chances of survival have improved considerably as well due to improved prevention, early detection, and treatment options (e.g., chemotherapy, radiation, operation, immunotherapy) [3]. The 10-year survival rate of malignant melanoma is remarkably high with 93-95% (men and women, respectively) [1]; leading to an increasing population of long-term malignant melanoma survivors (time since diagnosis ≥5 years). The long-term consequences for affected individuals, however, remain a significant concern, as the diagnosis itself as well as the intense treatments take their toll on individuals’ long-term physical and mental health. While the burden of cancer patients in acute treatment has been well-studied (with elevated rates of depression and anxiety) [4, 5], knowledge about long-term consequences for melanoma survivors is scarce [6].

Generally, cancer survivors face several challenges such as work-related issues [7], diminished quality of life, long-term and late treatment consequences [8, 9], eventually leading to persistently high prevalence rates of depression and anxiety even years after acute treatment [10–13]. A large meta-analysis [14] indicated a slightly increased risk of depression and anxiety in cancer survivors (pooled prevalence 21%, each) in comparison to the general population. Within survivors of malignant melanoma, elevated rates for depression and anxiety have been found 5 years after the diagnosis [15] as well as compared to the general population [10]; potentially indicating that not all cancer survivors have coped well with their disease. Although studies show that those with long-term psychological burden might be a minority [16], knowledge about what determines the coping process is scarce, especially among long-term survivors [6, 17]. To professionally support vulnerable cancer survivors, informed screening and intervention efforts are needed that target central risk and protective factors. Several researchers thus called for future studies to identify clinical and psychological determinants of long-term mental distress in cancer survivors generally [15] and specifically in malignant melanoma survivors [6].

### Optimism and pessimism’s link to mental health in individuals affected by cancer

Influencing the cognitive and emotional response to critical events as well as to everyday experiences [18, 19], optimism and pessimism, have repeatedly been linked to mental health outcomes in the general population [20] as well as in individuals with cancer [19]. Optimism and pessimism are defined as relatively stable characteristics shaping individuals’ expectancies for future outcomes [21]. Optimists are characterized by the fact that they have the inner conviction that future results will be positive for them, while pessimists assume a negative outcome [21]. As distinct dispositional outlooks, optimism and pessimism are consequential factors buffering or exacerbating the effects of stressors (e.g., cancer diagnosis) on mental health [20, 22].

For instance, an optimistic outlook co-occurs with fewer depressive symptoms, while pessimism has been found to increase those in individuals with cancer [e.g., 23–26]. In a recent meta-analysis, Fasano et al. [24] demonstrated that low levels of optimism predicted depression in individuals with cancer (pooled correlation: − 0.43). Optimism/pessimism also play a role in cancer patients’ anxiety symptoms [e.g., 27–31], with emerging evidence for its relevance in survivors as well [19]. In fact, some studies found optimism to predict anxiety symptoms but not depressive symptoms in individuals with cancer [28, 30]. Following Barlow et al. [32] optimism/pessimism might be crucial in developing anxiety by determining the individual response to uncertainty and uncontrollable situations [21].

### Coping strategies as mediator variable between optimism/pessimism and mental health

While optimism/pessimism can be conceptualized as more general, stable, and distal influences on mental health, coping styles are how these dispositions actually come to bear in daily life. Coping strategies are cognitive, emotional and behavioral responses to stress aiming to protect the self (e.g., self-esteem, required assumptions) and mitigate (emotional) stress [33]. Depending on their specific aim, coping strategies can, for instance, be categorized as *approach* vs. *avoidant* coping, or more specifically as *problem*-focused, *emotion*-focused and *avoidant* coping [34].

The use of approach (e.g., problem- or emotion-focused coping) or avoidant coping shapes cancer survivors’ mental health outcomes [35–38]. Longitudinal studies especially identified avoidant strategies as maladaptive in coping with cancer. For instance, in a study with 81 cancer survivors, avoidant coping predicted anxiety symptoms 10 years after diagnosis [39]. Bickel et al. [16] recently reported that depressive symptoms not only remained moderate to severe for some individuals even years after diagnosis, but also that reductions were associated with using less avoidant coping in a longitudinal study. Within survivors of malignant melanoma, the use of adequate coping strategies (e.g., active and cognitive coping) has been associated with fewer mental distress in a systematic review of 40 original studies [40]. Avoidance coping on the other hand seems to contribute to psychological morbidity among survivors of malignant melanoma [40].

The frequent usage of approach coping strategies, however, relies on the individual conviction of their effectiveness [41]. Optimism eventually influences positive expectancies of coping strategies when individuals face challenges [19]. If feeling overwhelmed or interpreting a challenge as unmanageable, using avoidant coping strategies becomes more likely [41]. Hence, optimism is positively associated with adaptive or approach (e.g., problem-focused coping, emotion-focused coping) and negatively with avoidance coping strategies [18, 19]. This link has been proposed since the 80s with Scheier & Carver [21] reporting that optimists are more likely to use active coping strategies or seek social support compared to their pessimistic counterparts. Further, they tend to be more flexible in their usage of coping strategies [41]. Especially in the event of less controllable challenges, such as cancer (survival), optimistic persons tend to use more adaptive coping strategies such as problem- or emotion-focused coping [19, 41].

Current research thus indicates an influence of both optimism/pessimism and coping strategies on mental health in those affected by cancer [24]. There are also indications that optimism/pessimism as more stable traits and coping strategies as more flexible characteristics seem to interact with each other [24]. Carver et al. [42] first suggested coping strategies to mediate the link between optimism and mental health outcomes, and Schou et al. [43] built on these findings: The authors longitudinally investigated 165 breast cancer patients and found that coping strategies mediated the effects of optimism/pessimism on quality of life one year after diagnosis.

### Study aim and hypotheses

As Bickel et al. [16] reported, most cancer patients’ mental distress decreases over time. However, not for all, and vulnerable survivors need professional support to effectively manage their symptoms or achieve relief from mental distress. Therefore, the present study aimed to contribute to a better understanding of individual – risk and protective – factors shaping mental health in the context of cancer survival.

Along these lines, the above-summarized evidence leaves a research gap: Despite multiple calls for research investigating the potentially mediating role that coping strategies play in the relationship between optimism/pessimism and mental health outcomes [e.g., 24, 36, 43], current research examining this interplay is still scarce, in particular in cancer *survivors*. Despite their increasing numbers, the majority of research has been conducted on cancer patients in the acute treatment phase. Furthermore, most studies were conducted with women affected by breast cancer [e.g., 35, 38, 39, 42] whose experience of the disease and survivorship trajectory might differ from those with other diagnoses [44]; in particular men [45]. Lastly, research in psycho-oncology has often heavily focused on mental health *risk* factors only [46].

Understanding addressable personal skills and protective factors, such as coping strategies, is crucial in informing psychosocial interventions to foster the prevention of mental distress as well as providing effective care for those who are still burdened years after diagnosis. The aim of the present work, thus, is to expand previous investigations of how coping strategies mediate the effect of optimism/pessimism on depressive and anxiety symptoms in cancer survivors (here, specifically, in long-term survivors of malignant melanoma). The following hypotheses and research questions guided this work:

1. Optimism and pessimism are associated with depressive and anxiety symptoms (optimism negatively; pessimism positively).
2. Active coping and seeking external support are associated with lower levels of depressive and anxiety symptoms.
3. The use of avoidant coping is associated with higher levels of depressive and anxiety symptoms.
4. Coping strategies mediate the link between optimism and depressive and anxiety symptoms, at least partly:
  - Optimism is positively associated with active coping and seeking external support and negatively associated with avoidant coping.
  - Pessimism is negatively associated with active coping and seeking external support and positively associated with avoidant coping.
5. In an exploratory manner, we also investigated whether associations of coping styles and optimism/ pessimism differ by gender?

## Methods

### Design, settings, and participants

The data was derived from the cancer registry of Rhineland-Palatinate at the University-Medical-Center Mainz, where all cancer patients are regularly registered by their physician. It includes anonymous personal identification and personal data (e.g., gender, age) of the cancer patient, cancer characteristics (e.g., tumour diagnosis (ICD-10), staging (TNM), time since diagnosis, patients’ identification data as well as data of the physician who registered the patient. The estimated completeness of incident melanoma notifications in Rhineland-Palatinate is ≥ 95%. In the current data, all cancer patients who were diagnosed and registered with malignant melanoma (ICD-10: C43) between 2000 and 2005 were included. Additional inclusion criteria were: (a) alive at assessment, (b) aged ≥ 14 years at diagnosis, and (c) available written informed consent. The data collection method implied that for all patients, time since diagnosis was ≥ 5 years. For legal reasons and confidentiality, cancer patients were not directly contacted by the study team, but rather dermatologists who registered potential cancer survivors were contacted. In the next step, cancer patients’ names were decoded by the cancer registry to enable physicians to identify study participants. Patients were forwarded the study information, written informed consent form and the questionnaire (via paper and pencil) by their dermatologist. Patients who did not react to the letter of interest within six weeks were sent a reminder. There were no further attempts in contacting potential participants. The recruitment was completed in May 2012.

In total, 75 out of 112 dermatologists handed forward the present study document. Non-participating dermatologists (*n* = 37, 33%) noted ‘no interest in studies’, ‘lack of time’, ‘no more patient contact’ or ‘no more medical practice’ for not participating. The participating dermatologists reached out to 1,702 former cancer patients, of which 382 could not be included due to being unavailable (*n* = 200, 11.8%), having died (*n* = 46, 2.7%), or exclusion by their dermatologist due to dementia or being unable to take part in the study (*n* = 136, 8%). Out of 1,320 successfully contacted patients, 689 (52.2%) could be included in the analysis. No further data is available for non-participating cancer survivors. The data was analysed without any reference to the personal identification. The protocol was approved by the Ethics Committee of the Statutory Physician Board of the State of Rhineland-Palatine (Reference number 837.161.11(7703)). It is described in detail in Beutel et al. [10]. The present work constitutes a secondary analysis of this data, adding new perspectives, to address the research gap described in the Introduction section.

### Measures

To capture the constructs of interest in a standardized way, established psychological questionnaires were employed whose German version had also been previously validated and proven to be psychometrically sound.

*Optimism and pessimism* were measured by the revised Life-Orientation-Test (LOT) [47]. Originally designed as a unidimensional tool, this revised questionnaire (LOT-R) consists of two subscales assessing generalised optimism and pessimism by 3 items each [48, 49]. Statements are rated on a Likert scale ranging from 0 = ‘strongly disagree’ to 4 = ‘strongly agree’. Internal consistency within the present sample was acceptable (optimism: *α* = 0.72; pessimism: *α* = 0.69).

*Coping Style* was assessed using the Brief Cope (BC) [50]. It consists of 14 scales with two items each. It uses a Likert scale ranging between 1 = ‘Never’ to 4 = ‘very often’. In line with previous investigations, we derived three scales using a factor-analytic procedure (see [10]) “Seeking External Support”, “Denial/Self-blame” (which includes avoidant coping), and “Active Coping”. All subscales reached an acceptable reliability (Seeking External Support: *α* = 0.75, Denial/Self-Blame: *α* = 0.74, Active Coping: *α* = 0.76) and accounted for 38% of the total variance [10].

*Depressive symptoms* were measured by the Patient Health Questionnaire depression module (PHQ-9) [51]. It assesses symptoms that occurred in the past two weeks. Response options range from 0 = ‘not at all’ to 3 = ‘nearly every day’. The scale’s sum score ranges from 0 to 27, with scores of 10 and higher representing clinical symptom burden [51]. Previous investigations have confirmed its good psychometric properties, reliability and validity [52]. Its internal consistency within the study sample was good (*α* = 0.86). Previous publications have examined the current samples’ depressive symptoms and compared it with the general population. Cancer survivors have demonstrated higher depressive symptoms (although their sum score was below the clinical cut-off) [10].

*Anxiety symptoms* were assessed by the General Anxiety Disorder Questionnaire (GAD-7) [53] which is a validated self-report scale. This questionnaire refers to symptom severity in the past two weeks. The response range for the 7 items ranges from 0 = ‘not at all’ to 3 = ‘nearly every day’. The scale’s sum score ranges from 0 to 21, with scores of 10 and higher representing clinical symptom burden [53]. Internal consistency in the current sample was good (*α* = 0.89). Again, Beutel et al. [10] previously compared anxiety symptoms of the current sample with those of the general population. Here, only female but not male cancer survivors reported greater anxiety [10].

### Data analysis

**Fig. 1:**
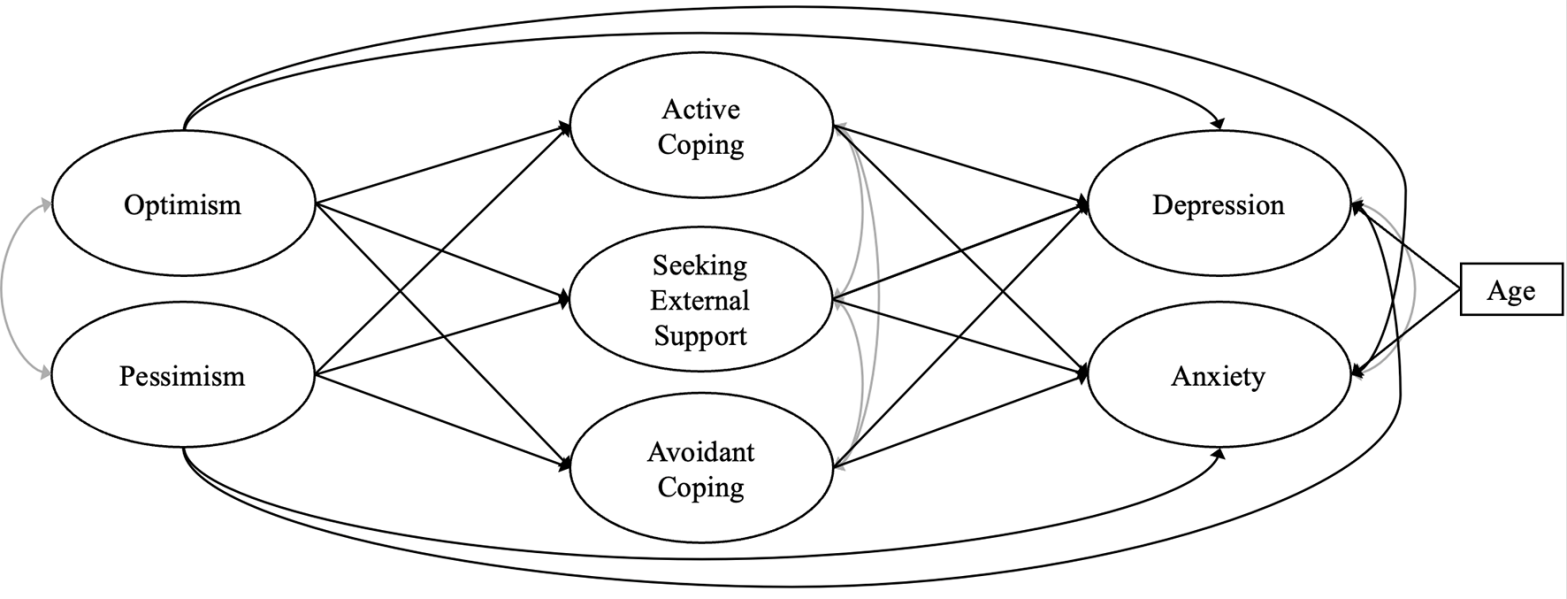
Proposed model of coping strategies (incl. active coping, seeking external support, avoidant coping) mediating the effect of optimism/pessimism on depression and anxiety in cancer patients

Data were analysed using R version 4.3.1. To describe socio-demographic characteristics, patients’ coping strategies and symptoms, descriptive statistics (e.g., mean, variance) were calculated. Pearson correlations were used to evaluate the association between age, optimism/pessimism, coping styles, depressive and anxiety symptoms. For group comparisons, t-/welch’s-tests were conducted with consideration of potential violations of assumptions. To test the hypothesized mediation, a theoretically derived structural equation model was performed. The proposed mediation model is displayed in Fig. 1. We included the variables of interest as well as age (as a covariate) if they were correlated with the dependent variables. We tested the model using the Satorra-Bentler Correction to handle non-normally distributed data [54, 55]. To assess model fit the following goodness of fit indices and cut-offs were applied: Comparative Fit Index (CFI) and Tucker-Lewis-Index (TLI) values ≥ 0.95, Root Mean Square Error of Approximation (RMSEA), Standardized Root Mean Square Residual (SRMR) ≤ 0.08 [56, 57]. We also conducted a sensitivity analysis in women and men to test the generalizability of the observed patterns.

## Results

### Sample characteristics

The current sample is almost balanced in terms of gender (women: 51.38%, men: 48.62%), and included cancer survivors with an age range of 28 to 94. The participants reported time since the cancer diagnosis ranged from 5.67 to 12.17 years.

Further sample characteristics are shown in Table 1.

**Table 1:** (Socio-)Demographic characteristics of cancer survivors (*N* = 689)

	Total sample	Women (*n* = 354)	Men (*n* = 335)
	*N* (%)	*N* (%)	*N* (%)
Age (in years)	61.71 (14.57)	58.62 (15.17)	64.98 (13.16)
< 30	6 (0.87)	4 (1.13)	2 (2.10)
30–39	46 (6.68)	30 (8.47)	16 (4.78)
40–49	112 (16.26)	84 (23.73)	28 (8.36)
50–59	136 (19.74)	79 (22.32)	57 (17.01)
60–69	136 (19.74)	57 (16.10)	79 (23.58)
> 70	253 (36.72)	100 (28.25)	153 (45.67)
Educational level			
Primary school	333 (48.40)	154 (43.50)	179 (53.59)
Secondary school	196 (28.49)	124 (35.03)	72 (21.56)
High school	142 (20.64)	71 (20.06)	71 (21.26)
Other	17 (2.47)	5 (1.41)	12 (3.60)
Marital status ^a^			
Single	49 (7.12)	30 (8.47)	19 (5.69)
Married	520 (75.58)	242 (68.36)	278 (83.23)
Separated/divorced	49 (7.12)	29 (8.19)	20 (5.99)
Widowed	70 (10.17)	53 (14.97)	17 (5.09)
Cancer stage at time of diagnosis (UICC) ^b^			
Stage I	365 (52.98)	197 (55.65)	168 (50.16)
Stage II	34 (4.94)	13 (3.67)	21 (6.27)
Stage III	7 (1.02)	3 (0.85)	4 (1.19)
Stage IV	0 (0.0)	0 (0.00)	0 (0.00)
Unknown	283 (41.07)	141 (39.83)	142 (42.39)
Time since diagnosis ^c, d^	9.00 (2.81)	8.81 (2.80)	9.20 (2.81)

Note. ^a^ data of one person missing; ^b^ UICC-Stage: For year of diagnosis up to 2003 according to TNM 5th edition (German version, Springer Verlag 1997), for year of diagnosis 2004 or later according to TNM 6th edition (German version, Springer Verlag 2002); Subdivisions according to TNM 6 in A or B were summarized in respective stages; ^c^ in years; ^d^ M (SD)

### Correlations and group comparison

In the *full sample*, depressive and anxiety symptoms were significantly correlated with all variables of interest (including age); but in different directions. Seeking external support and active coping were neither correlated with optimism nor pessimism. Denial/self-blame was correlated with optimism and pessimism. In *women*, depression was associated with optimism and pessimism as well as denial/self-blame, but not with active coping, seeking external support or age. Anxiety was associated with optimism and pessimism, denial/self-blame, and active coping. No relevant link was seen between anxiety and seeking external support or age. Optimism and pessimism were associated with all coping strategies. In *men*, depression and anxiety were both associated with optimism/ pessimism, and all coping strategies and age. Optimism and pessimism were associated with denial/self-blame age, not with active coping and seeking external support. The full correlation matrix is shown in Table 2.

We compared men and women regarding the constructs of interest, including the symptom measures (Table 3). In the present sample, women reported higher levels of both depression and anxiety symptoms. Further, women reported less optimism and more usage of active coping and external support strategies than men.

**Table 2:** Correlation of mental health variables

				Coping				
	Age	Optimism	Pessimism	SES	Active Coping	Denial/ Self-Blame	Depression	Anxiety
*Total sample*								
Age	1							
Optimism	-137***	1						
Pessimism	0.090*	− 0.335***	1					
SES	− 0.071	0.002	− 0.058	1				
Active Coping	− 0.234***	0.055	− 0.030	0.439***	1			
Denial/ Self-blame	− 0.079*	− 0.315***	0.330**	0.168***	0.317***	1		
Depression	− 0.110**	− 0.446***	0.334***	0.102***	0.163***	0.478***	1	
Anxiety	− 0.164***	− 0.428***	0.303***	0.115***	0.179***	0.485***	0.803***	1
Women (*n* = 354)								
Age	1							
Optimism	0.104	1						
Pessimism	0.055	0.414***	1					
SES	0.026	0.114*	− 0.112*	1				
Active Coping	− 0.136*	0.188***	− 0.127*	0.391***	1			
Denial/ Self-blame	− 0.071	− 0.285***	0.389***	0.102	0.266***	1		
Depression	− 0.032	− 0.395***	0.369***	0.023	0.104	0.420***	1	
Anxiety	− 0.093	− 0.398***	0.343***	0.035	0.133*	0.426***	0.780***	1
Men (*n* = 335)								
Age	1							
Optimism	0.129*	1						
Pessimism	0.126*	− 0.261***	1					
SES	− 0.075	− 0.063	0.007	1				
Active Coping	− 0.271***	− 0.033	0.077	0.435***	1			
Denial/ Self-blame	− 0.065	− 0.338***	0.279***	0.219***	0.356***	1		
Depression	− 0.157**	− 0.489***	0.306***	0.138*	0.184***	0.539***	1	
Anxiety	− 0.187***	− 0.440***	0.277***	0.138*	0.177**	0.546***	0.825***	1

Note. SES = Seeking External Support; * = *p* <.05, ** = *p* <.01, *** = *p* <.001

**Table 3:** Gender differences in optimism/pessimism, the use of coping mechanisms and mental distress

	Mean (SD)			Test statistics		
	Full sample	Women	Men	T(df)	95% CI	*p*
Coping						
SES	1.96 (0.61)	2.10 (0.61)	1.81 (0.57)	*t*(682) = -6.32	-0.38; -0.20	**< 0.001**
Active Coping	2.07 (0.55)	2.18 (0.54)	1.96 (0.54)	*t*(681) = -5.32	-0.30; -0.14	**< 0.001**
Denial/Self-Blame	1.32 (0.37)	1.34 (0.35)	1.30 (0.39)	*t*(681) = -1.44	-0.10; 0.01	0.150
Optimism	3.04 (0.84)	2.95 (0.85)	3.14 (0.82)	*t*(680) = 3.07	0.07; 0.32	**0.002**
Pessimism	1.59 (0.92)	1.57 (0.92)	1.61 (0.93)	*t*(678) = 0.57	-0.10; 0.18	0.567
Depression	3.86 (4.03)	4.26 (4.19)	3.43 (3.81)	*t*(687) = -2.71	-1.43; -0.23	**0.007**
Anxiety	3.34 (3.77)	3.85 (3.90)	2.80 (3.54)	*t*(685.83) = -3.70	-1.61; -0.49	**< 0.001**

Note. SD = standard deviation; CI = Confidence Interval; SES = Seeking External Support; Range of Seeking External Support, Active Coping, Denial/Self-Blame 1–4; Range of Optimism/Pessimism 0–4; Range of Depression 0–27; Range of Anxiety 0–21. Test statistics for the differences by gender. Significant differences are indicated in bold

### Main analyses

#### Full sample

**Fig. 2:**
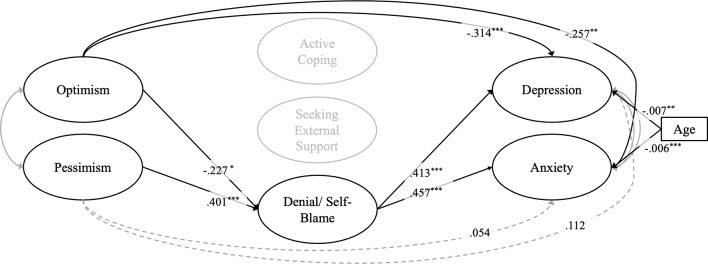
Visualization of the mediation model of the effects of optimism/pessimism on depressive and anxiety symptoms via denial/self-blame within the full sample (adjusted for age). The figure shows the standardised estimates for the model. Statistically significant associations are indicated by drawn-through black lines. Nonsignificant associations are depicted by dashed lines. Optimism but not pessimism had a direct link to depressive and anxiety symptoms. Both optimism and pessimism show an indirect effect via denial/self-blame on depression and anxiety symptoms, respectively. The effect of pessimism on depression and anxiety was fully mediated by denial/self-blame coping

In the full sample, only denial/self-blame was included as potential mediating coping strategy (following the results of the correlation analysis). We also included age as a covariate. The model fit was excellent (CFI = 0.917, TLI = 0.909, RMSEA = 0.048, SRMR = 0.052). Within this model, optimism - but not pessimism - showed statistically significant *direct* (negative) effects on depression and anxiety symptoms. Optimism and pessimism showed an *indirect* effect on both depressive and anxiety symptoms via denial/self-blame. The indirect effect of *optimism – denial/self-blame – depression* explained 22.87% of the total effect of optimism on depression. The indirect effect of *pessimism – denial/self-blame – depression* explained 60.80% of the total effect of optimism on depression. The indirect effect of *optimism– denial/self-blame– anxiety* explained 28.21% of the total effect of optimism on depression. The indirect effect of *pessimism– denial/self-blame– anxiety* explained 79.55% of the total effect of optimism on depression. Altogether, the model accounted for 50.20% of the variance in depressive and 45.10% of the variance in anxiety symptoms in women. The structural model for the mediation model and its standardized effects are shown in Fig. 2. Detailed results are reported in Table 4.

**Table 4:** Results of the full-sample mediation analysis of optimism/pessimism on depressive and anxiety symptoms via denial/self-blame

	Statistical prediction of			
	Depressive symptoms		Anxiety symptoms	
	ß	*p*	ß	*p*
Direct effects				
Age	− 0.003	**0.006**	− 0.006	**< 0.001**
Optimism	− 0.196	**< 0.001**	− 0.190	**< 0.001**
Pessimism	0.072	0.141	0.041	0.460
Denial/Self-Blame	0.647	**< 0.001**	0.848	**< 0.001**
Indirect effects				
Optimism via Denial/Self-Blame	− 0.059	**0.022**	− 0.077	**0.017**
Pessimism via Denial/Self-Blame	0.107	**0.001**	0.140	**0.001**
Total Effects				
Optimism	− 0.258	**< 0.001**	− 0.273	**< 0.001**
Pessimism	0.176	**< 0.001**	0.176	**0.004**

Note. SES = Seeking External Support; Statistically significant paths/effects are indicated in bold

#### Models stratified by gender

In the following, we report on the stratified models, starting with the *male subsample*: Again, only denial/self-blame was included as potential mediating coping strategy. We also included age as a covariate. The model fit was good (CFI = 0.904, TLI = 0.894, RMSEA = 0.051, SRMR = 0.062). Optimism showed a *direct* link to depressive (*ß* = − 0.156, *p* =.008), but not anxiety symptoms. No *indirect* effect was found for optimism, neither on depressive nor anxiety symptoms. Pessimism showed *indirect* links with depressive and anxiety symptoms via denial/self-blame (pessimism– denial/self-blame– depression: *ß* = 0.131, *p* =.009; pessimism– denial/self-blame– anxiety: *ß* = 0.163, *p* =.011). The indirect effect of pessimism via denial/self-blame accounted for 58.22% of the total effect of pessimism on depressive and for 65.99% of the total effect of anxiety symptoms in men. In total, the model accounted for 62.90% of the variance in depressive and 54.1% of the variance in anxiety symptoms in men. Findings are reported in detail in the supplement material (Table S1, Fig. S1).

In *women*, we included denial/self-blame as a potential moderator for the link between optimism/pessimism and depressive symptoms and added active coping for the link between optimism/pessimism with anxiety symptoms (in concordance with previous correlation analysis). Age was not included as a covariate as it did not show relevant links to the variables of interest. The model fit within the female subsample was good (CFI = 0.878, TLI = 0.868, RMSEA = 0.047, SRMR = 0.076). Optimism was *directly* associated with depressive (*ß* = − 0.293, *p* =.003) and anxiety symptoms (*ß* = − 0.346, *p* =.025). While optimism was linked to active coping, no *indirect* effect on depressive or anxiety symptoms was found. Pessimism did not show *direct* associations with depressive or anxiety symptoms but was *indirectly* associated with depressive symptoms via denial/self-blame (*ß* = 0.177, *p* =.021). Contrasting indirect and total effects as above, the indirect effect of pessimism on depressive symptoms via denial/self-blame accounted for 145.08% of the total effect, indicating a *suppression effect* also referred to as inconsistent mediation [58]. This was because of the combination of mediators that included potential risk as well as protective factors alongside each other, and the observed associations thus have different polarities: the direct association of pessimism on depressive symptoms was negative (*ß* = − 0.054, *p* =.598) and although it was nonsignificant, it was included in the calculation of the total effect. By contrast, the indirect path via denial/self-blame had positive values, with the opposite signs thus constituting a suppression of the total effect. Altogether, the model accounted for 42.00% of the variance in depressive and 41.00% of the variance in anxiety symptoms in women. Findings are reported in detail in the supplement material (Table S2, Figure S2).

## Discussion

The present work aimed to shed light on the role of coping strategies in the context of psychological distress in cancer survivors. More precisely, we examined whether active coping, seeking external support and avoidant coping mediated the link between optimism/pessimism with depressive and anxiety symptoms and also investigated whether the coping process may be different for male versus female cancer survivors. The overarching aim was to derive (gender-sensitive) actionable insights for prevention and intervention in clinical practice, i.e., targets for psychosocial interventions. This was the first study analyzing these specific pathways of possible mental distress evolvement in long-term cancer survivors.

The present findings emphasize optimism, a relatively stable personality trait [21], as a robust, independent protective factor against psychological distress in cancer survivors. Consistent with previous research, we found that optimism was directly associated with depressive [23, 24] and anxiety symptoms [28, 30]; regardless of the employed coping strategies. Solberg Nes & Segerstrom [41] concluded in their meta-analysis that optimists tend to adjust their coping strategies to the individual situation. As the long-term burden of cancer can vary greatly over time, these stressors might rather call for flexible coping to maintain and recover mental health than for one dominant one.

However, gender-stratified analyses revealed that this generally resilient effect of optimism may only be valid in *female* cancer survivors. While those reported a significant link of optimism with both depressive and anxiety symptoms, respectively, this effect was not as strong in *male* cancer survivors: Optimism was directly associated with depressive symptoms (although more weakly than in women), but no direct or indirect link was found with anxiety symptoms. This aligns with previous research, specifically finding the link between optimism and anxiety symptoms to be specific for female cancer survivors [19]. Moreover, when comparing gender-stratified correlations of optimism/pessimism, coping strategies and depressive and anxiety symptoms, different patterns emerged. While in female cancer survivors, approach coping strategies (active coping and seeking external support) were linked to optimism and pessimism but not to depressive and anxiety symptoms, the opposite was observed in men. Here, coping strategies were associated with depressive and anxiety symptoms but not with optimism and pessimism. Approach coping strategies may thus not be as efficient in protecting against adverse mental health outcomes. Rather, protective dispositions (e.g., optimism) may act as general resilience factors in female cancer survivors. Additionally, women may tend to opt for coping strategies relying on their general disposition rather than due to the individual situation; possibly hampering flexible coping due to specific stressors. A recent systematic review also described this paradox of female cancer patients choosing *more* positive coping strategies but, at the same time, reporting *more* psychological distress; emphasizing the insufficient effectiveness of the applied coping strategies [59]. Male cancer survivors, on the other hand, seem to choose their coping strategies more flexibly and less associated with their general disposition; potentially enabling more individualized problem-solving and reduced psychological distress.

However, the described gender effect is reversed regarding the dispositional *risk* factor pessimism. Again, depressive symptoms are somewhat similarly affected by an indirect effect of pessimism (risk disposition) via avoidant coping for both, male and female cancer survivors. Concerning anxiety, however, this indirect effect was *only* found in men. Pessimistic men tend to avoid problems and negative emotions and thus not only increase the risk of depressive but also anxiety symptoms. At the same time, pessimism is also linked to avoidant coping in women, but these strategies were only associated with depressive not anxiety symptoms.

Findings thus suggest different emotional pathways for the emergence of anxiety symptoms in female and male cancer survivors; with (the lack of) protective dispositions being central in female and avoidant coping strategies being central in male cancer survivors.

While previous research has emphasized gender-dependent emotional processing in the context of cancer [59, 60], findings are still scarce and inconsistent [59]. Nonetheless, the present findings are somewhat in line with previous research. Optimism was more strongly associated with anxiety symptoms in female than male cancer survivors [19], and emerging evidence indicates gender-specific differences in the association between avoidant coping strategies and depressive and anxiety symptoms in cancer patients [60]. While in women, avoidant coping strategies were only associated with depression, they were also linked with anxiety in men [60].

Generally, men tend to rely more on avoidant coping then women (who relied more on social support) [59, 60]. This may be (partly) due to gender role socialization and the consequently reduced self-disclosure in men [61]. Conformity to traditionally masculine norms such as self-reliance and emotion suppression [62] could thus amplify the negative effects of avoidant coping on overcoming the long-term stress of cancer [63]. As they perhaps struggle to cope with more difficult feelings, avoidant coping seems to be especially central in pessimistic men (who might also be pessimistic about the potential outcomes of self-disclosure or accessing social support). Thus, although the present work investigates the associations of interest in an individualized manner, i.e., looking at participants’ dispositions and coping strategies, a cultural/community perspective is needed as the gender norms an individual internalizes and conforms to are shaped by the society around them. Along these lines, previous research has highlighted the ways in which the communication around cancer survival has oftentimes focused on women and neglected male experiences [64]. This phenomenon begins with terminology and continues with a persistent gap in research on the feelings and experiences of men with cancer [45], but this can also lead to practical consequences, e.g., men might be less frequently approached (also by healthcare professionals) about their potential mental distress.

### Limitations

When interpreting these findings, the following limitations must be considered: First, we must critically note that the stratified models explained a comparatively larger proportion of variance in both depression and anxiety symptoms in men compared to women (depression: 62.90% vs. 42.00%; anxiety: 54.10% vs. 41%). This suggests that influencing factors of particular relevance to depression and anxiety in women may have not been as well represented. Future studies thus might not just replicate current findings but may also extend the model by including potential factors that are associated with female coping. Second, the present study is of cross-sectional nature, which does not allow for causal conclusions. As cancer survivors are a comparatively understudied population with regard to the interplay of individual differences and mental health, this work expanded the available evidence base of how they cope in the long-term and which individual factors are relevant in this process, but more research is needed examining cancer survivors’ adaption and mental health trajectory in longitudinal designs. Second, the data was restricted to cancer survivors of malignant melanoma. Despite the knowledge of the differential influence of specific cancer entities [e.g., 43, 65], this limits the generalizability. However, we opted for this specific sample in order to rule out possible interference effects due to the cancer diagnosis. Additionally, due to the frequency of diagnosis combined with the remarkably high survival rate [1], survivors of malignant melanoma are a large population, which has previously been described as burdened [e.g.,^ 10^, ^15^]. Third, data was collected in 2012. Since then, there were diverse (social) events and changes that could have had implications for cancer survivors’ well-being and coping (i.e., COVID-19, potential shifts in the population’s views and attitudes towards cancer (survivorship)). Future studies thus need to replicate and extend current findings by also including other cognitive factors associated with the studied variables (e.g., self-efficacy, perceived control). Finally, the response proportion of this study was relatively low (32,6%). Due to registry-based data and the applicable legal regulations, participants were contacted indirectly by their former physician with a relatively long follow-up period. While registry-based data harbors advantages (e.g., > 95% of melanoma patients are registered) the sampling procedure does not allow for specific conclusions about the cancer survivors who did not take part. However, rudimentary comparisons were conducted elsewhere [66]. Results indicated slight differences in age and sex (i.e. non-participants were older, and more likely to be female) but not regarding other socio-demographic or cancer characteristics [66]. Lastly, the observed differences between men and women could only be interpreted theoretically with regard to potential gender norms and norm-conforming behavior. Future research should explicitly assess participants’ convictions and attitudes regarding such norms and their internalization and conformity. We also lacked information to contextualize participants’ current symptom levels and coping within their life circumstances (beyond SES, age, etc.), and age was only included as a potentially confounding variable, limiting the present findings’ integration with relevant lifespan theories (e.g., socioemotional selectivity theory [67]).

## Conclusion

While optimism does not come to bear via specific coping strategies, pessimism does. The present findings show that the effect of pessimism on depression (and anxiety) symptoms was fully mediated by avoidant coping strategies. However, these associations showed partial differences in anxiety symptoms in male versus female cancer patients and, therefore, lead to gender-sensitive clinical implications. Our findings highlight the pivotal role of optimism and pessimism as dispositional factors influencing long-term psychological distress in cancer survivors, while also including coping strategies as potential mediators of this process.

Optimism emerged as a general protective factor, particularly for female cancer survivors, being directly linked to both depressive and anxiety symptoms. In male cancer survivors, optimism was linked to depressive symptoms but showed no direct or indirect associations with symptoms. Findings thus suggest that the resilient effect of optimism may be more pronounced in women, whereas men might be more affected by risk factors. Pessimistic men may be at increased risk for both depressive and anxiety symptoms, whereas in women, the adverse indirect effect primarily elevated depressive symptoms.

Findings thus suggest the need for gender-sensitive psychosocial interventions tailored to male and female cancer survivors’ distinct coping mechanisms. As vulnerable *female* cancer survivors may opt for inefficient approach coping strategies, psychosocial interventions should aim to enhance their effectiveness rather than merely encourage women to use them. Generally, interventions could strengthen optimistic thinking patterns and foster a positive future outlook, possibly by integrating cognitive-behavioral approaches, promoting optimism as well as greater coping flexibility. For *male* survivors, interventions should target the adverse effects of avoidant coping and support them in facilitating approach coping strategies. Additionally, the stigmatization and taboos surrounding men’s mental health should be reduced. This certainly includes conducting more studies including gender-sensitive analyses when investigating emotional processes in the context of cancer (survival); but also in the context of other life challenges.

## Electronic supplementary material

Below is the link to the electronic supplementary material.

Supplementary Material 1

## Data Availability

The dataset presented is not readily available because the written informed consent of the study participants is not suitable for public access to the data and this concept was not approved by the local data protection officer and ethics committee. Supplemental material (incl. the R-code of the reported analyses) is provided via Open Science Framework: https://osf.io/fh7b4/?view_only=9e21634beed943a394cd62830aefa16a.

## Abbreviations

BC: Brief COPE
CFI: Comparative Fit Index
CI: Confidence Interval
GAD-7: Generalized Anxiety Disorder scale (7-item version)
ICD-10: International Classification of Diseases, 10th Revision
LOT: Life Orientation Test
LOT-R: Life Orientation Test Revised
M: Mean
PHQ-9: Patient Health Questionnaire (9-item version)
RMSEA: Root Mean Square Error of Approximation
SD: Standard Deviation
SES: Seeking External Support
SRMR: Standardized Root Mean Square Residual
TLI: Tucker Lewis Index
TNM: Tumor Node Metastasis (Cancer Staging System)
UICC: Union for International Cancer Control
