# Supplementary material for: The interplay of coping styles and optimism/pessimism in shaping mental health in long-term survivors of malignant melanoma: a register-based cohort study
Source: BMC Psychol. 2025 Apr 12;13:376. doi: 10.1186/s40359-025-02704-1 (PMC11993944; doi:10.1186/s40359-025-02704-1)
Supplement: Supplementary file 1 — Supplementary Material 1 [file 40359_2025_2704_MOESM1_ESM.docx]

**SUPPLEMENT MATERIAL**

| **Table S1.** Results of the mediation analysis of optimism/pessimism on   depressive and anxiety symptoms via coping styles within the male sample. | ……. 2 |
| --- | --- |
| **Figure S1.** Visualization of the mediation model of the effects of   optimism/pessimism on depressi*on* and anxiety via denial/self-blame in   men (adjusted for age). | ……. 3 |
| **Table S2.** Results of the mediation analysis of optimism/pessimism on   depressive and anxiety symptoms via coping styles within the female   sample. | ……. 4 |
| **Figure S2.** Visualization of the mediation model of the effects of   optimism/pessimism on depression and anxiety via coping styles in   women. | ……. 5 |

**Table S1.** Results of the mediation analysis of optimism/pessimism on depressive and anxiety symptoms via coping styles within the male sample.

|  | | Statistical prediction of | | | | | | |
| --- | --- | --- | --- | --- | --- | --- | --- | --- |
|  |  | Depressive symptoms | | | Anxiety symptoms | | | |
|  | | *ß* | *p* | | *ß* | | | *p* |
| Direct effects | |  | |  | | |  | |
|  | Age | - .004 | **.002** | | - .006 | | | **< .001** |
|  | Optimism | - .156 | **.008** | | -.125 | | | .067 |
|  | Pessimism | .098 | .090 | | .091 | | | .193 |
|  | Denial/Self-Blame | .679 | **< .001** | | .840 | | | **< .001** |
| Indirect effects | |  | | |  | | | |
|  | Optimism via  Denial/Self-Blame | - .055 | .147 | | - .069 | | | .136 |
|  | Pessimism via  Denial/Self-Blame | .131 | **.009** | | .163 | | | **.011** |
| Total Effects | |  | | |  | | | |
|  | Optimism | - .216 | **.001** | | - .200 | | | **.009** |
|  | Pessimism | .225 | **.001** | | .247 | | | **.009** |
| *Note*. SES = Seeking External Support; Statistically significant paths/effects are indicated in bold. | | | | | |  |  |  |

**
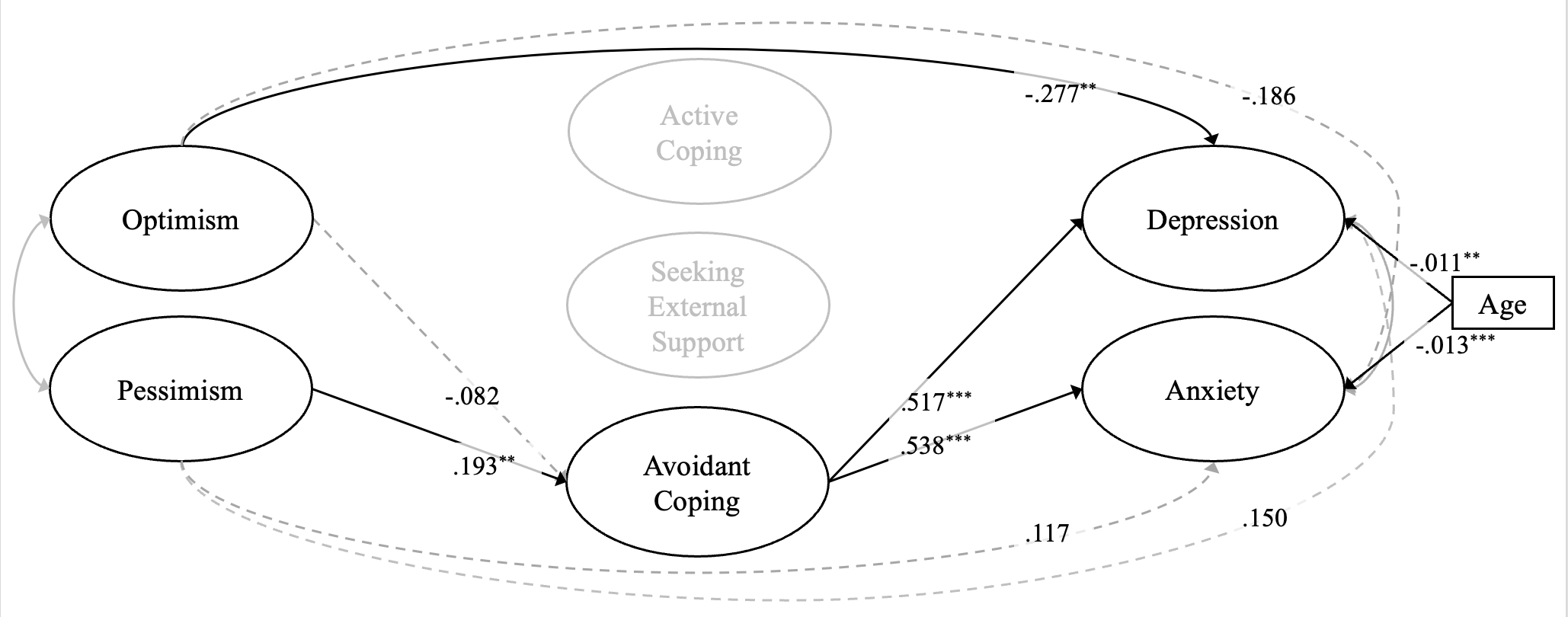
Figure S1.** Visualization of the mediation model of the effects of optimism/pessimism on depression and anxiety via denial/self-blame in men (adjusted for age).

The figure shows the standardised estimates for the model. Statistically significant associations are indicated by drawn-through black lines. Nonsignificant associations are depicted by dashed lines. Optimism had a direct effect on depression but not anxiety. Pessimism did not show a direct effect on neither depression nor anxiety, but showed indirect effects on both via denial/self-blame. Reported associations refer to the male subsample.

**Table S2.** Results of the mediation analysis of optimism/pessimism on depressive and anxiety symptoms via coping styles within the female sample.

|  | | Statistical prediction of | | | | | | |
| --- | --- | --- | --- | --- | --- | --- | --- | --- |
|  |  | Depressive symptoms | | | Anxiety symptoms | | | |
|  | | *ß* | *p* | | *ß* | | | *p* |
| Direct effects | |  | |  | | |  | |
|  | Optimism | -.293 | **.003** | | -.346 | | | **.025** |
|  | Pessimism | -.054 | .598 | | -.101 | | | .481 |
|  | Active Coping | - | - | | .064 | | | .808 |
|  | Denial/Self-Blame | .847 | **.008** | | 1.064 | | | .128 |
| Indirect effects | |  | | |  | | | |
|  | Optimism via  Active Coping | - | - | | .021 | | | .809 |
|  | Optimism via  Denial/Self-Blame | -.006 | .900 | | -.008 | | | .899 |
|  | Pessimism via  Active Coping | - | - | | .008 | | | .811 |
|  | Pessimism via  Denial/Self-Blame | .177 | **.021** | | .222 | | | .151 |
| Total Effects | |  | | |  | | | |
|  | Optimism | -.299 | **.003** | | -.344 | | | **.006** |
|  | Pessimism | .122 | .160 | | .129 | | | .207 |
| *Note*. SES = Seeking External Support; Statistically significant paths/effects are indicated in bold. | | | | | |  |  |  |

**
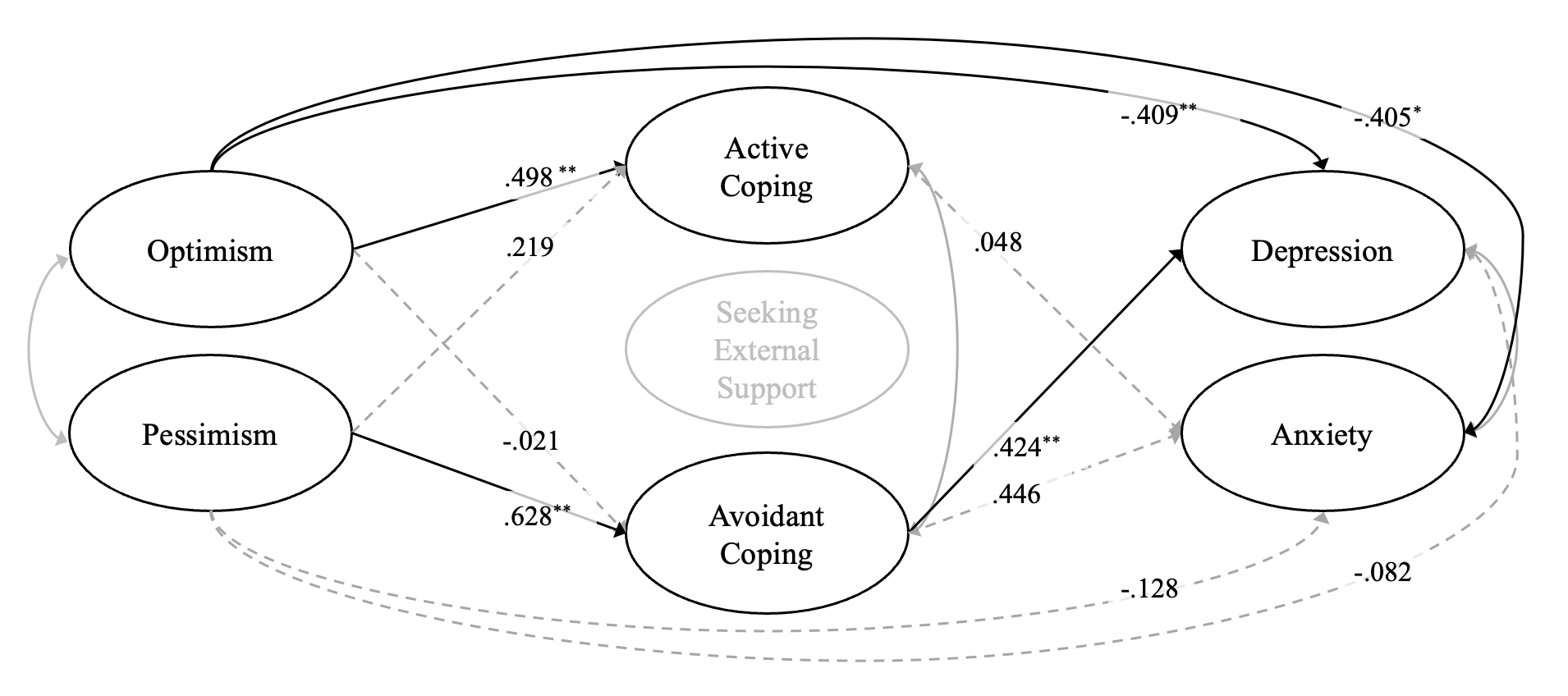
**

**Figure S2.** Visualization of the mediation model of the effects of optimism/pessimism on depression and anxiety via coping styles in women.

The figure shows the standardised estimates for the model. Statistically significant associations are indicated by drawn-through black lines. Nonsignificant associations are depicted by dashed lines. Optimism had a direct link to depression and anxiety. While Optimism was related to active coping, no indirect effect on depression and anxiety was found. Pessimism did not show a direct association with depression and anxiety but was indirectly linked to depression via denial/self-blame. Reported associations refer to the female subsample.
